# Supplementary material for: Parameters Influencing Moisture Diffusion in Epoxy-Based Materials during Hygrothermal Ageing—A Review by Statistical Analysis
Source: Polymers (Basel). 2022 Jul 12;14(14):2832. doi: 10.3390/polym14142832 (PMC9325284; doi:10.3390/polym14142832)

Figure S1. List of epoxy prepolymers in the study.

Name and details	Chemical structure
DGEBA<br>Bisphenol A diglycidylether	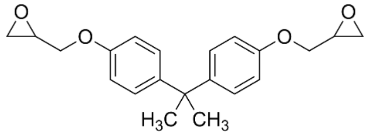
DGEBF<br>Bisphenol F diglycidylether	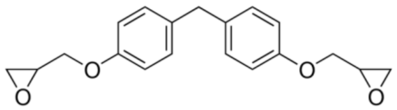
DGEBD<br>Butanediol diglycidyl ether	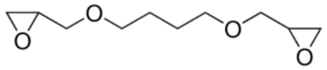
ESO<br>Epoxidized soybean oil	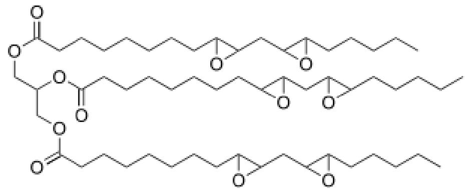
mTGAP<br>m-triglycidylaminophenol	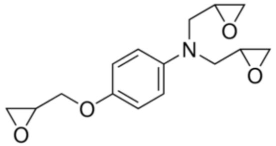

Name and details	Chemical structure
Novolac epoxy	Example: Epoxidised cresol novolac<br>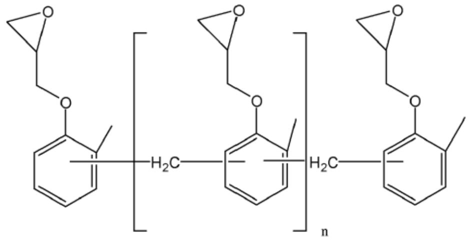
Rubber-modified epoxy	-
TCDAM<br>Diaminotetrachlorodiphenylmethane	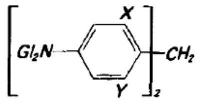
TGDDM<br>Tetraglycidyl methylenedianiline	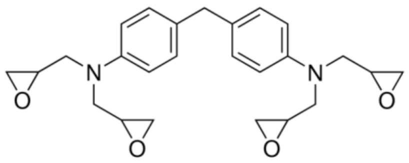

Figure S2. List of hardeners in the study (1/2).

Group	Name and details	Chemical structure
Aliphatic amines	DETA<br>Diethylenetriamine	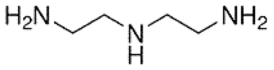
	TETA<br>Triethylenetetramine	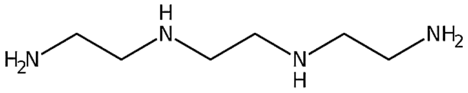
	EDA<br>Ethylene diamine	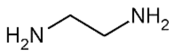
	Polyoxypropylenediamine	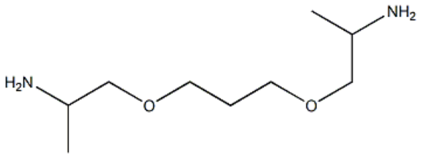
	TAEA<br>Trisaminoethylamine	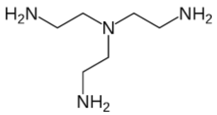
Cycloaliphatic amines	IPDA<br>Isophorone diamine	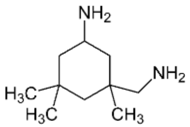
	DACH<br>Diaminocyclohexane	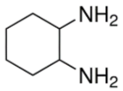
	Aminoimidazoline	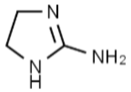

Group	Name and details	Chemical structure
Aromatic amines	DDS<br>Diaminodiphenylsulfone	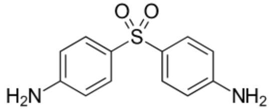
	DDM<br>Diaminodiphenylmethane	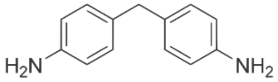
	MPD<br>Diaminobenzene	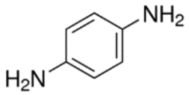
	MDEA<br>Methylenebisdiethylaniline	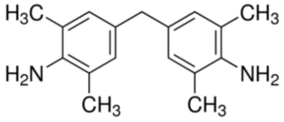
	M-MIPA<br>Methylenebisisopropylmethylaniline	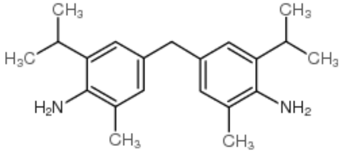
	Aniline	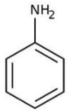
	DETDA<br>Diethyltoluenediamine	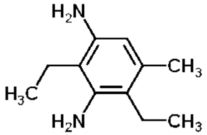
	mPDA<br>Metaphenylenediamine	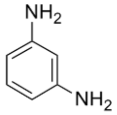
	POEA<br>Polyoxyalkyleneamine	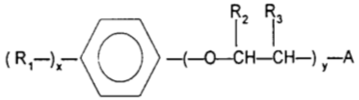
	Xylylene amine	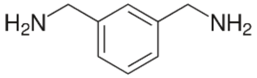

Figure S2. List of hardeners in the study (2/2).

Group	Name and details	Chemical structure
Anhydride acid	<u>Dibenzol peroxide</u>	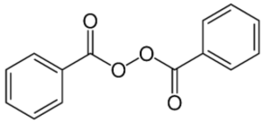
	<u>MHHPA</u><br>Methyltetrahydrophthalic anhydride	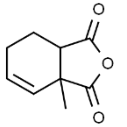
	<u>MNA</u><br>Methylnorbornenedicarboxylic anhydride	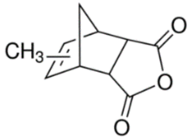
Dicyan-diamide	<u>DDA</u><br>Dicyandiamide	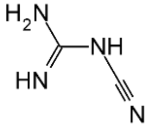
Amido amine	<u>Amidoamine</u>	Example: Lauramidopropyldimethylamine<br>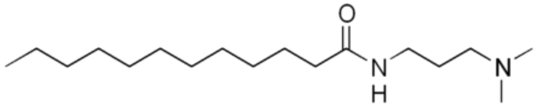
Phenol Novolac	<u>Phenol Novolac</u>	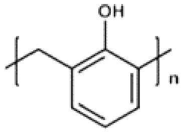 Example: Phenol-formaldehyde novolac

Figure S3. Boxplots of *D* as a function of epoxy prepolymer type.

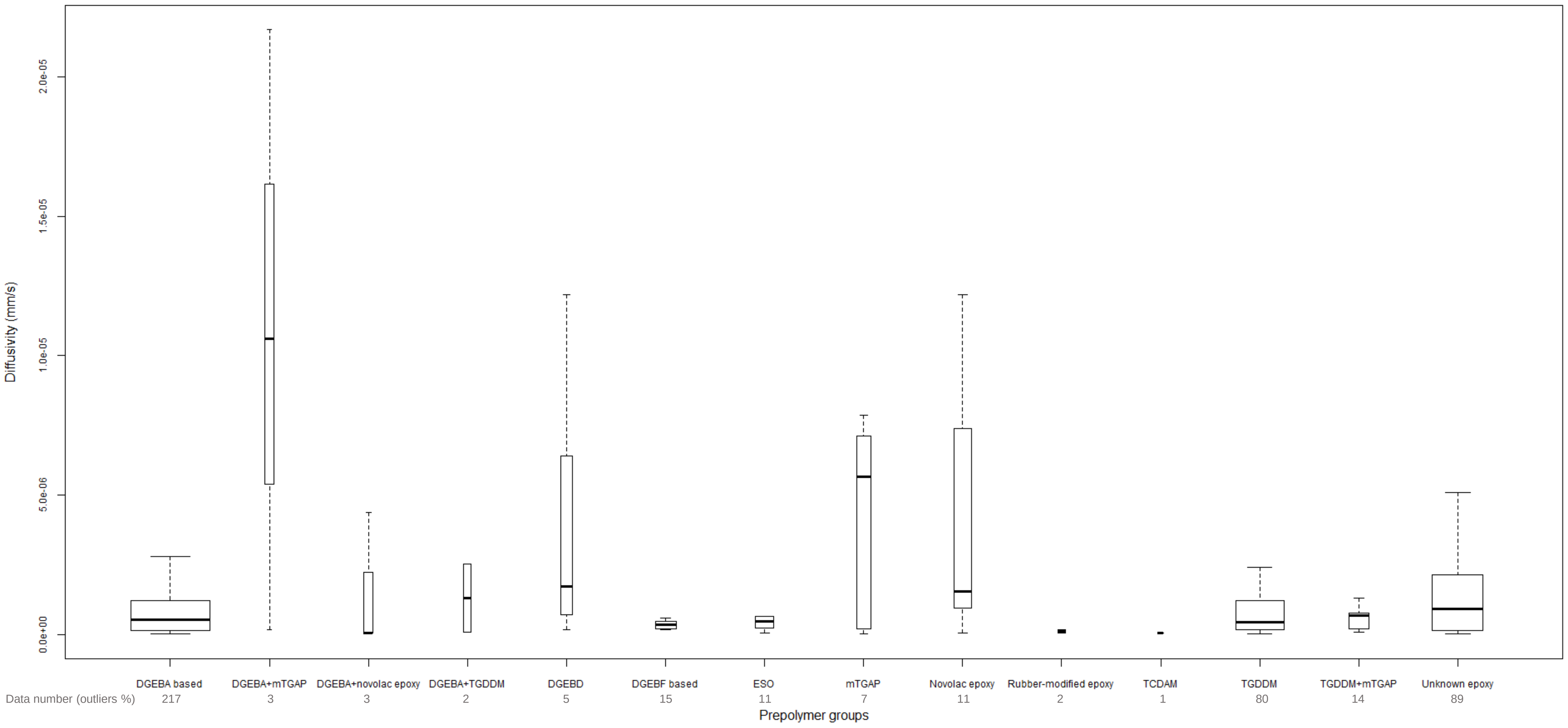

Figure S4. Boxplots of t_{sat}/h as a function of epoxy prepolymer type.

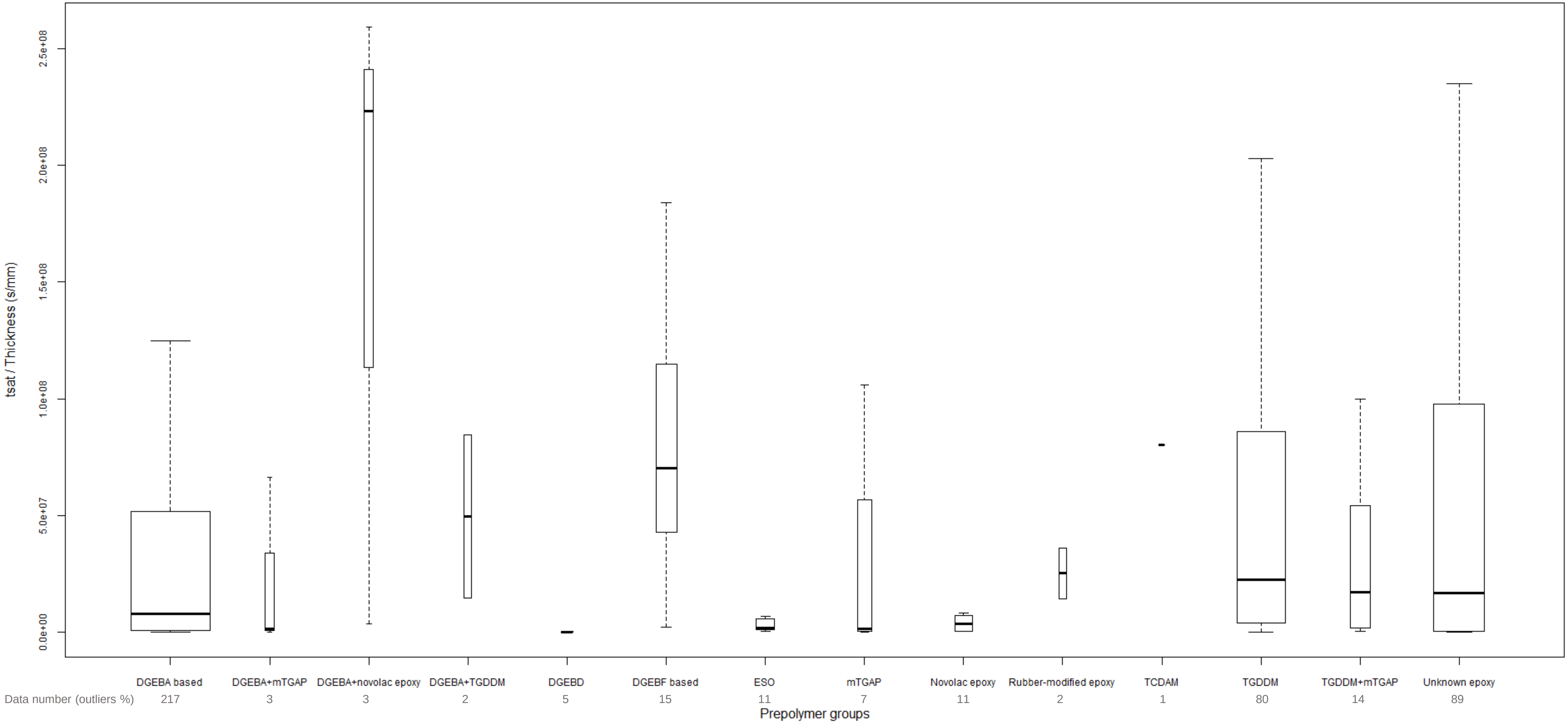

Figure S5. Boxplots of M_{sat} as a function of epoxy prepolymer type.

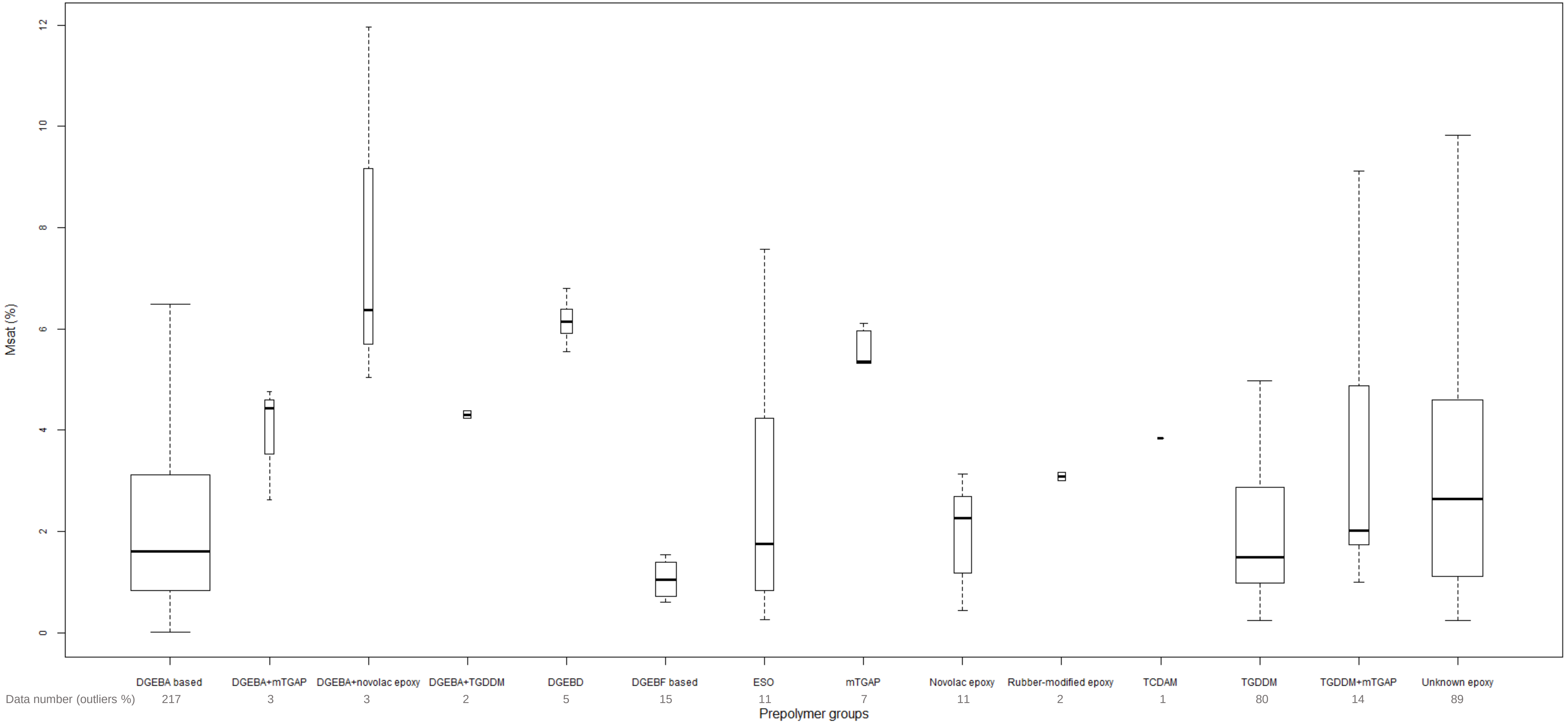

Figure S6. Boxplots of D as a function of hardener type.

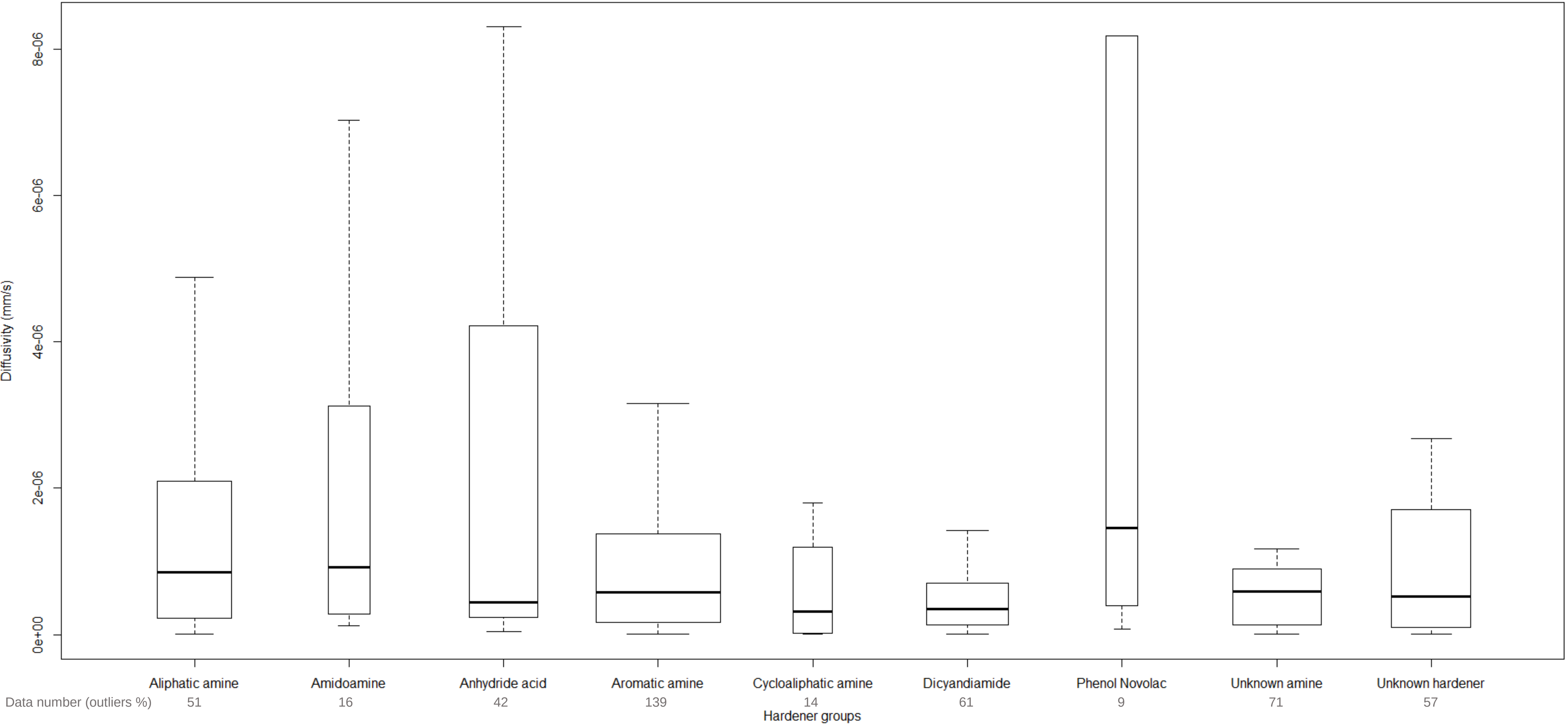

Figure S7. Boxplots of t_{sat}/h as a function of hardener type.

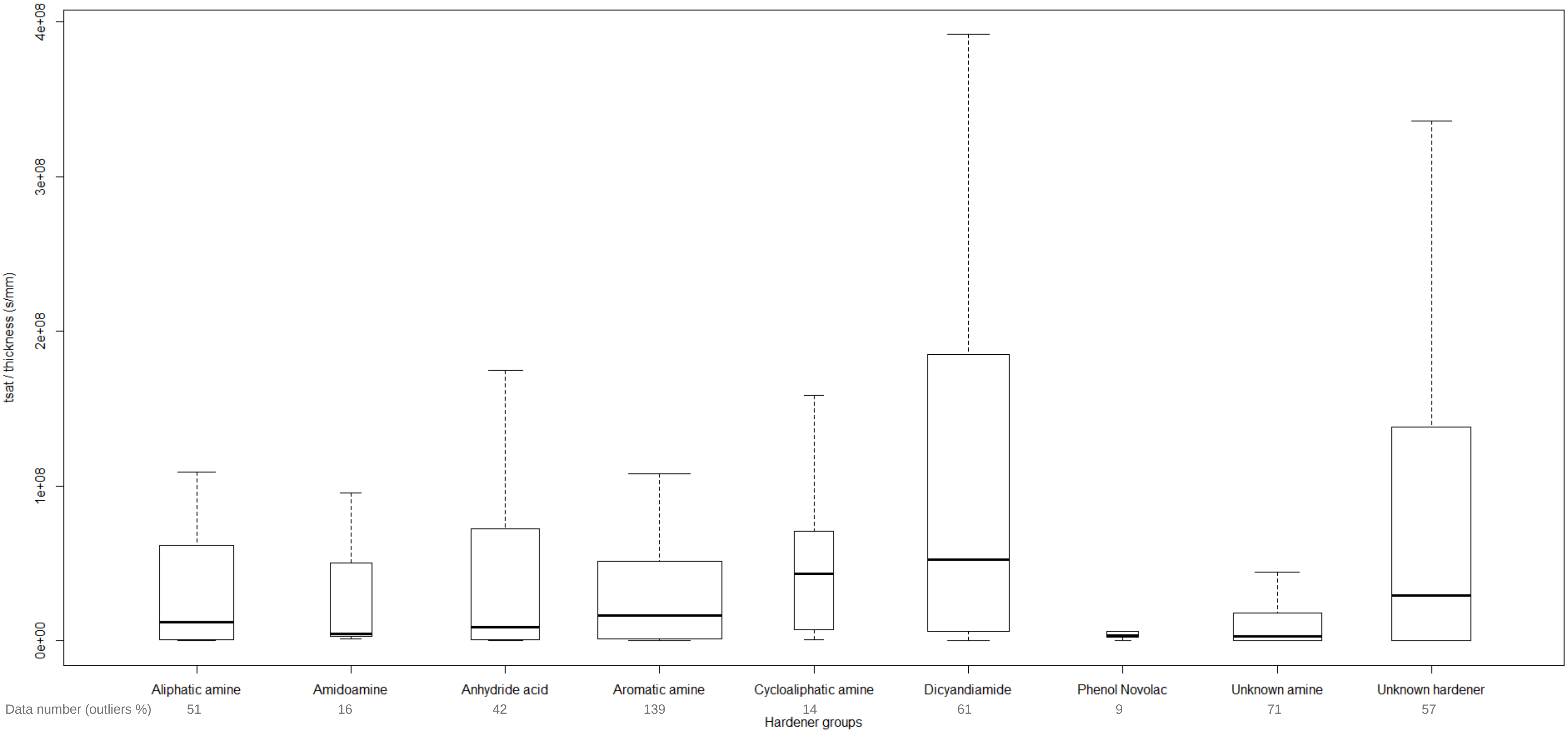

Figure S8. Boxplots of M_{sat} as a function of hardener type.

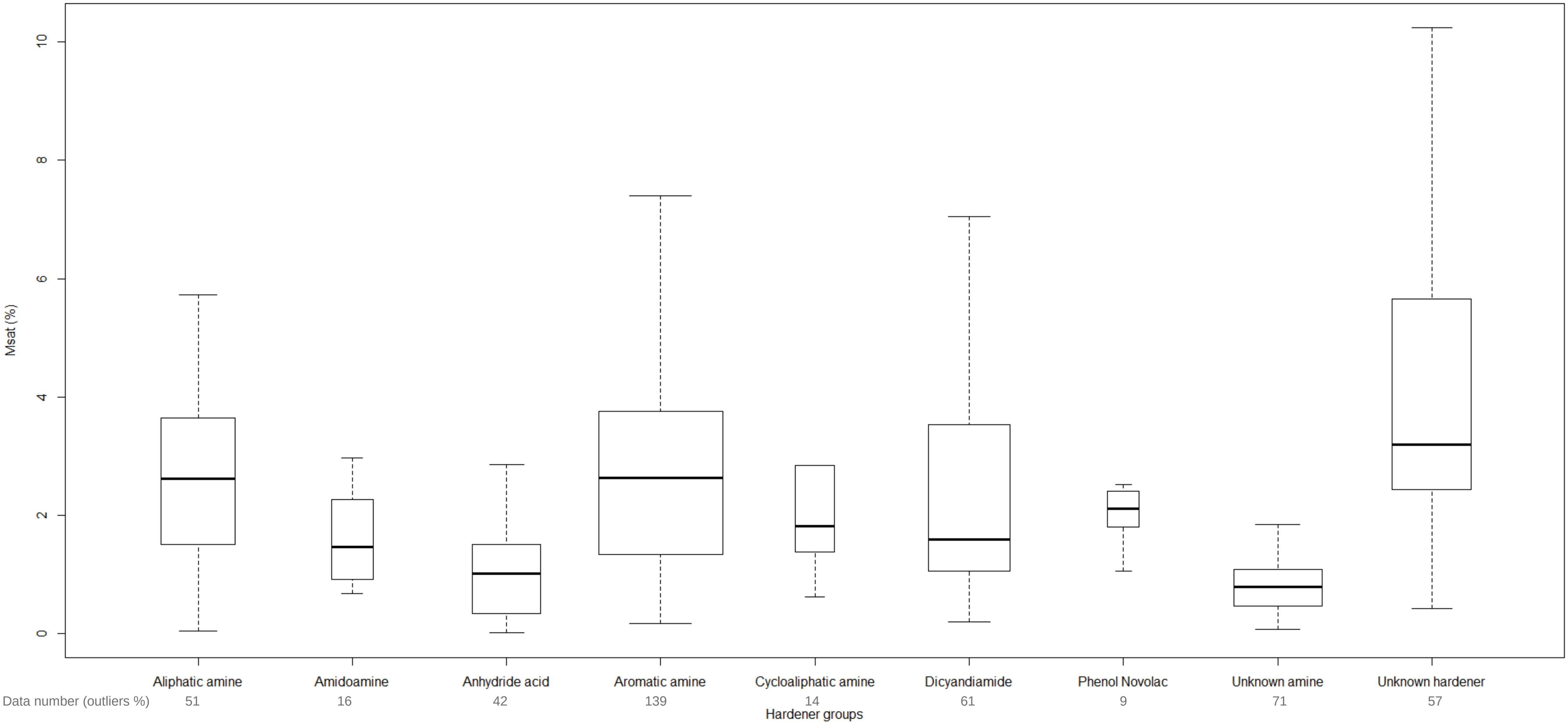

Figure S9. Boxplots of D as a function of reinforcement type.

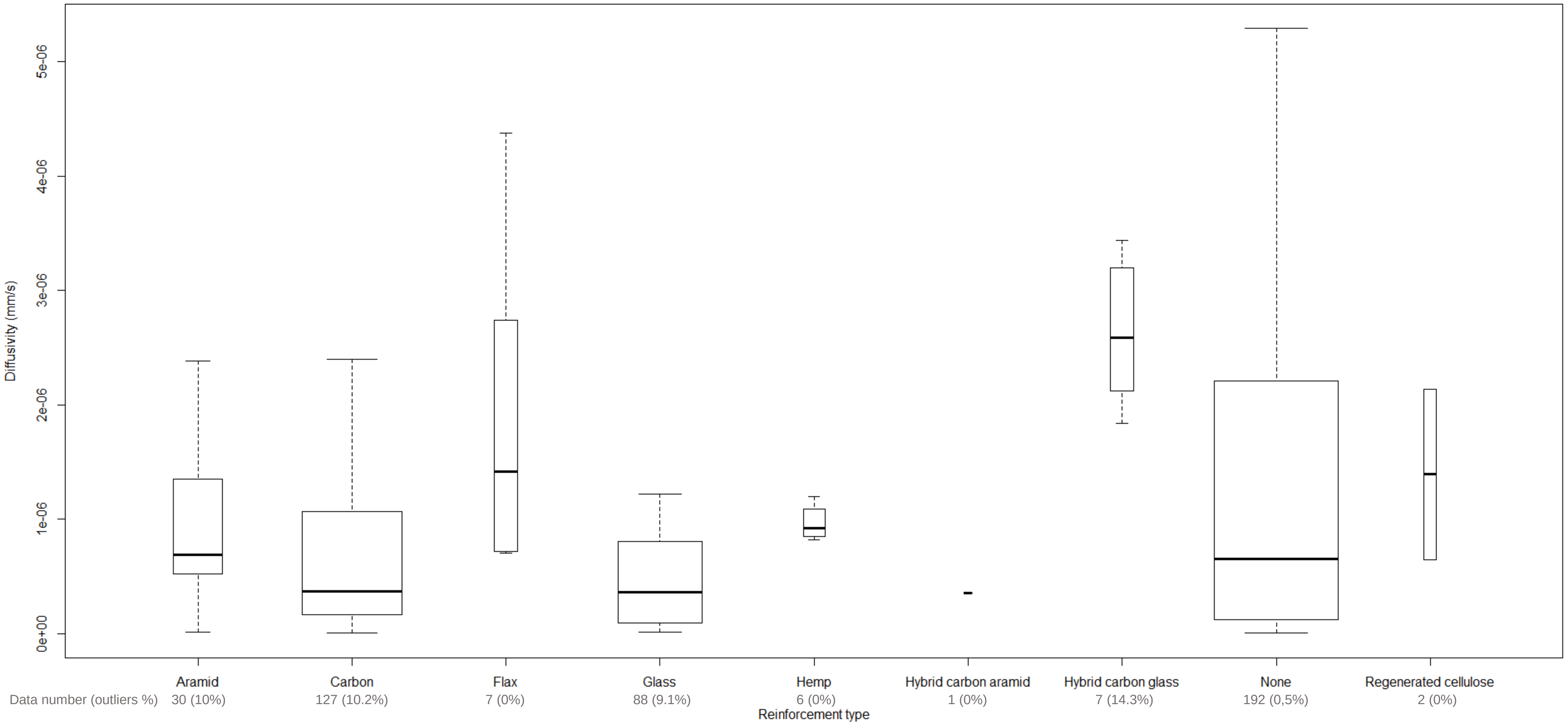

Figure S10. Boxplots of M_{sat} as a function of reinforcement type.

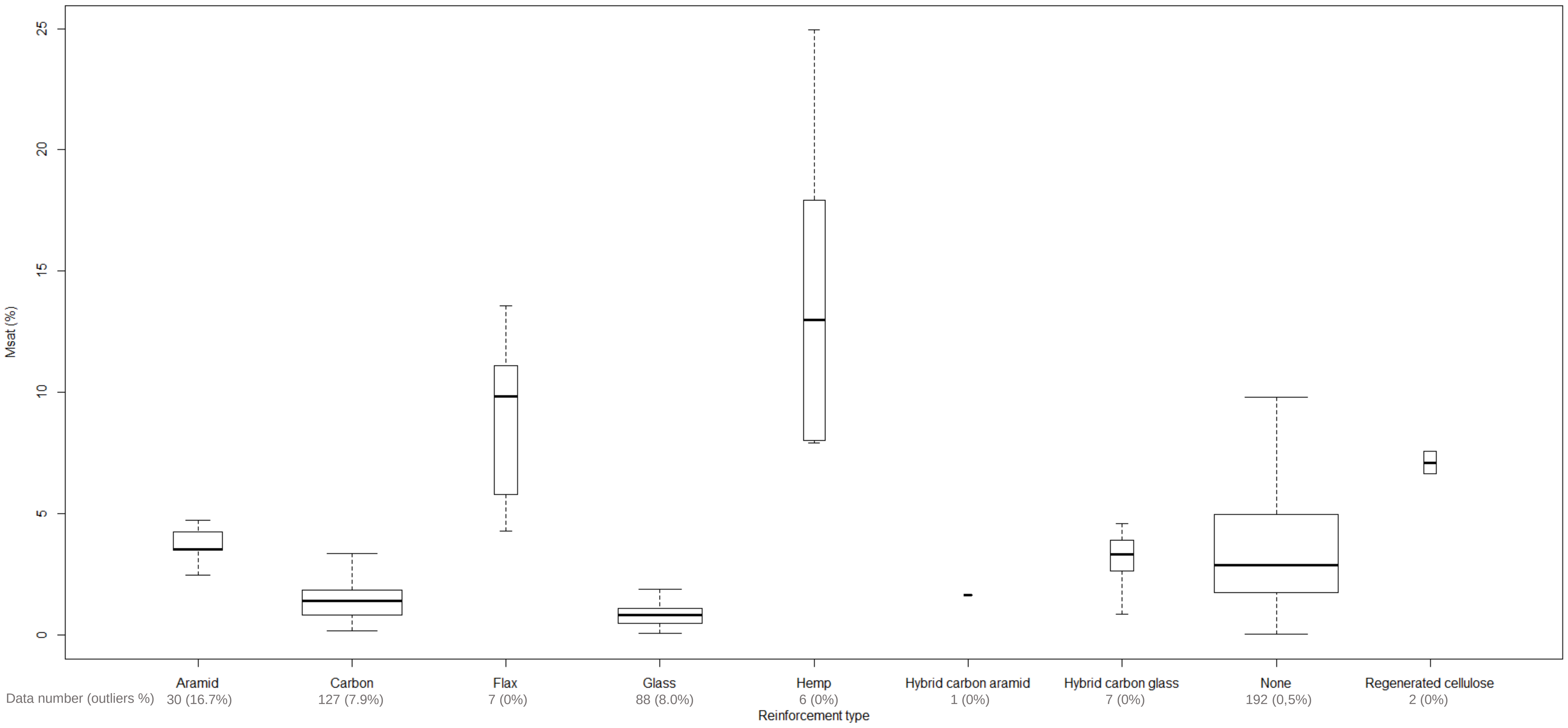

Figure S11. Boxplots of t_{sat}/h as a function of reinforcement type.

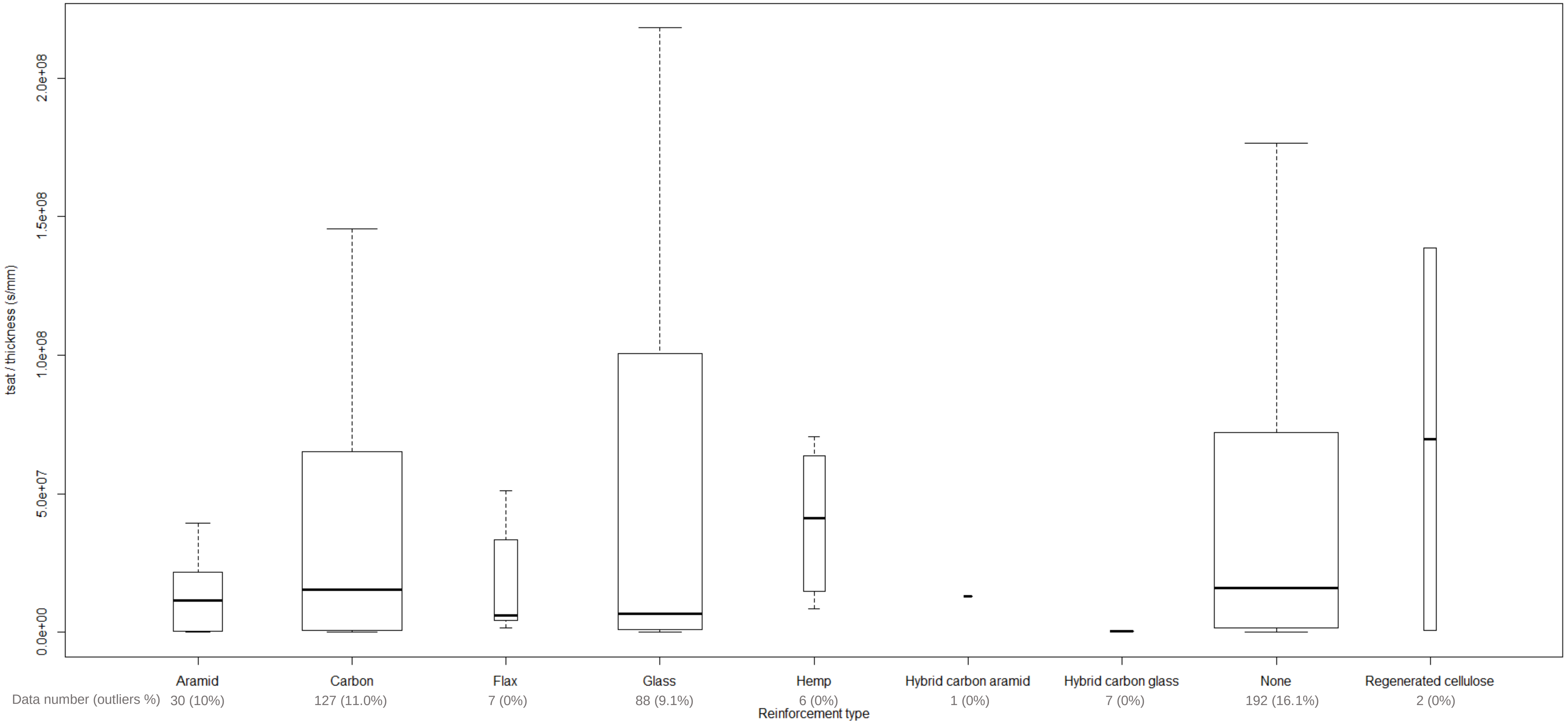

Figure S12. Boxplots of *D* as a function of fibre architecture.

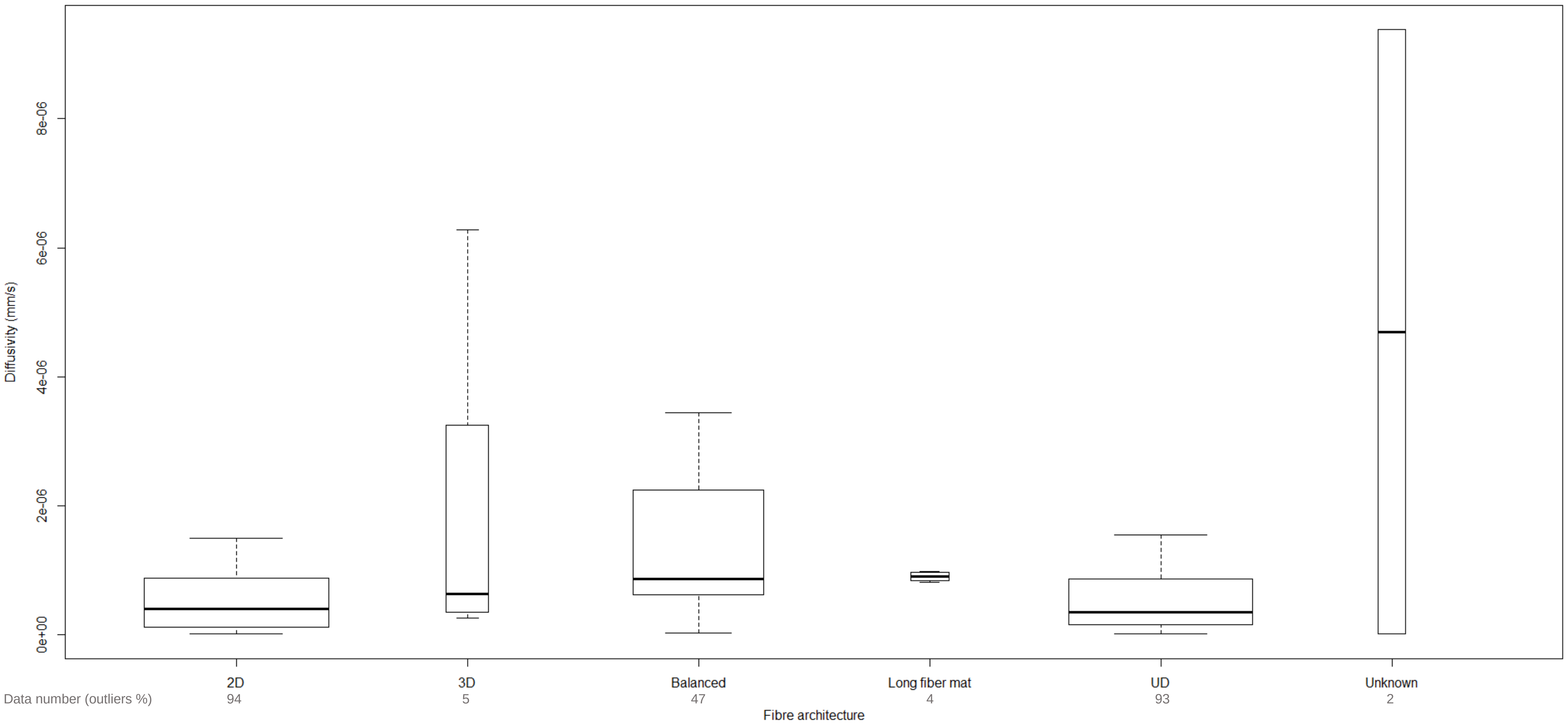

Figure S13. Boxplots of t_{sat}/h as a function of fibre architecture.

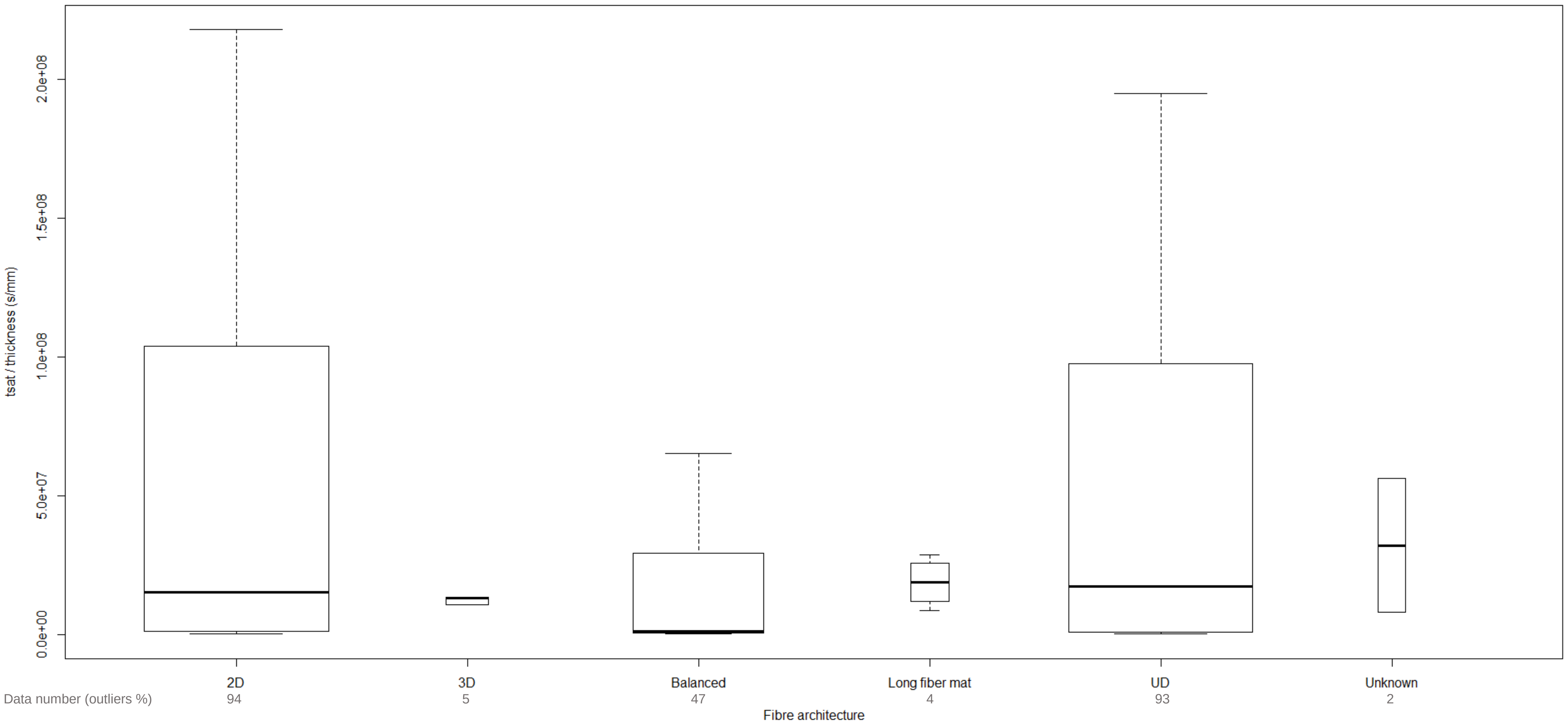

Figure S14. Boxplots of M_{sat} as a function of fibre architecture.

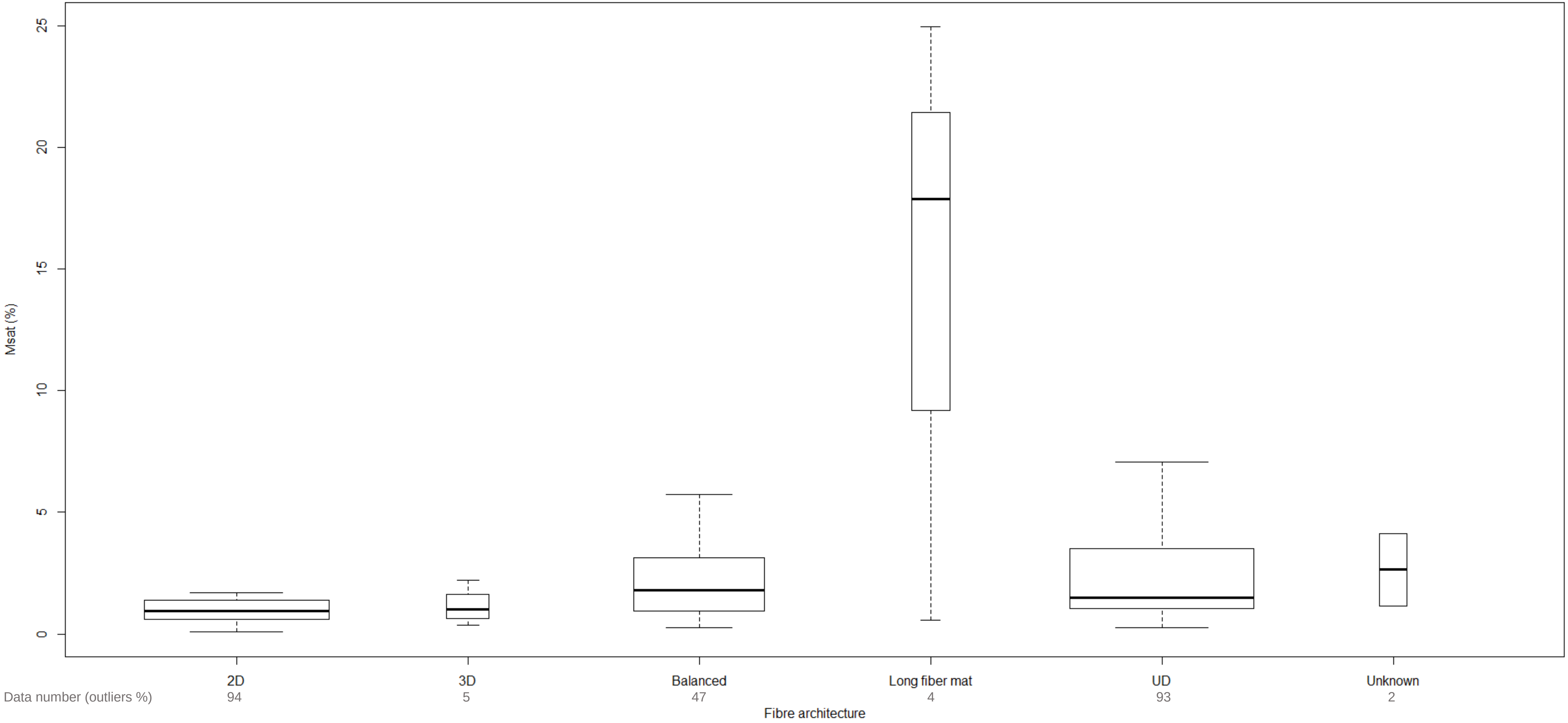

Figure S15. Boxplots of *D* as a function of process.

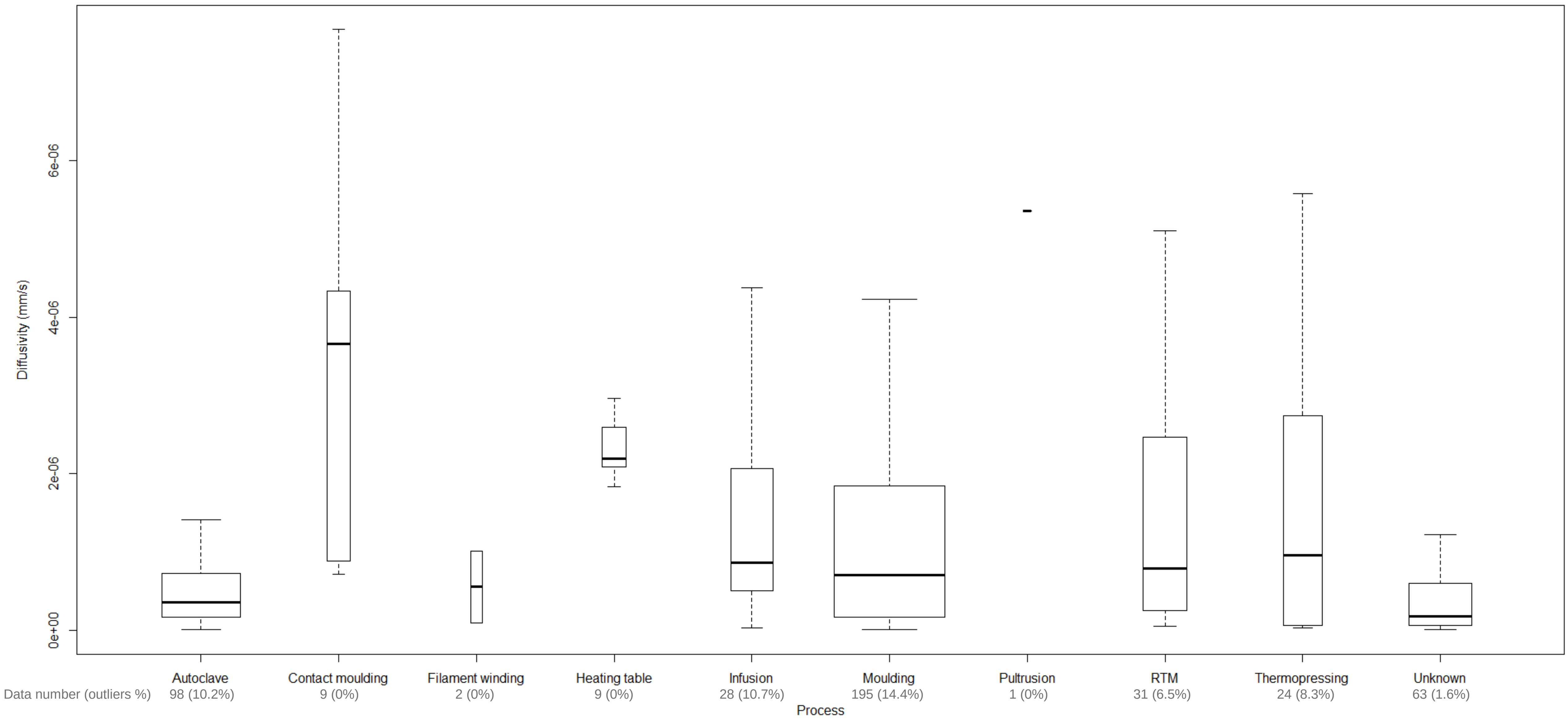

Figure S16. Boxplots of t_{sat}/h as a function of process.

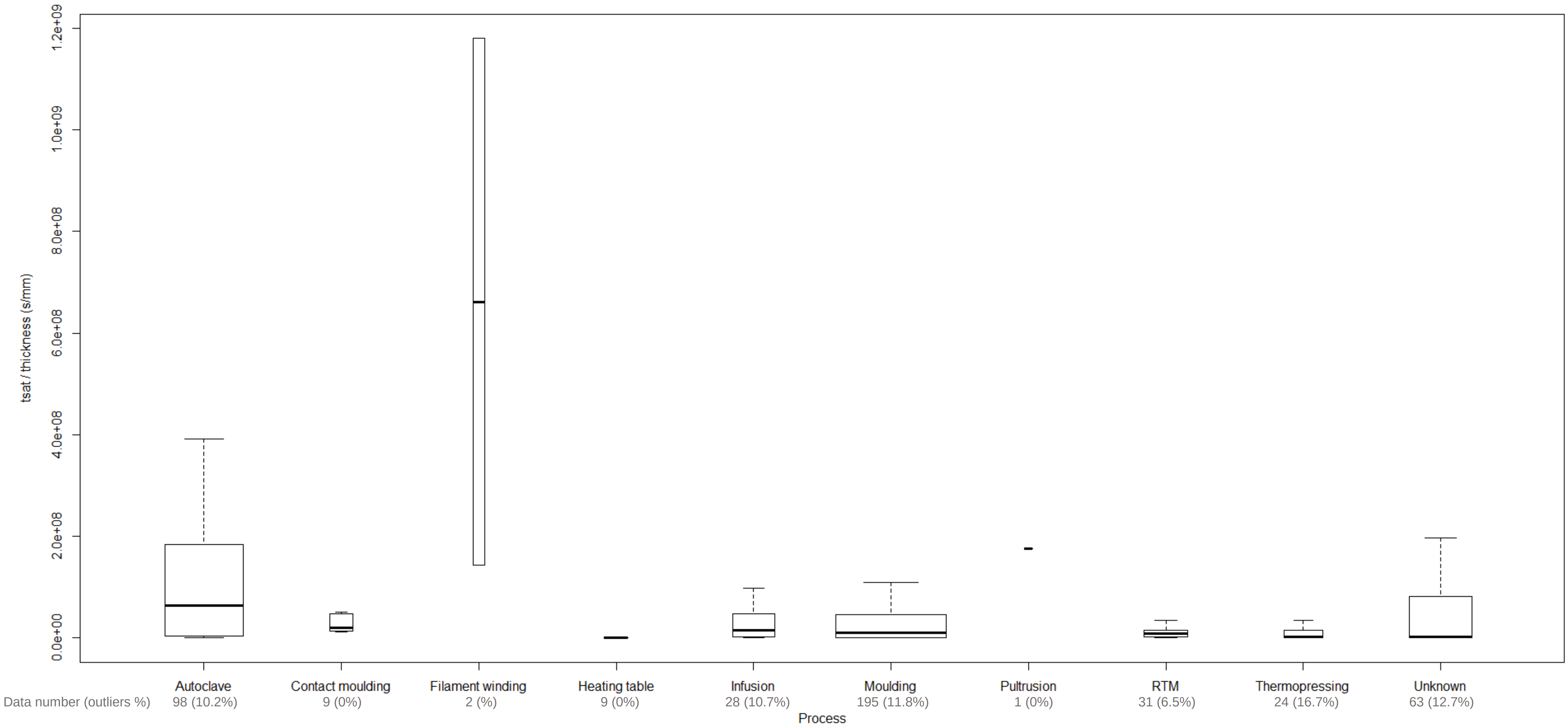

Figure S17. Boxplots of M_{sat} as a function of process.

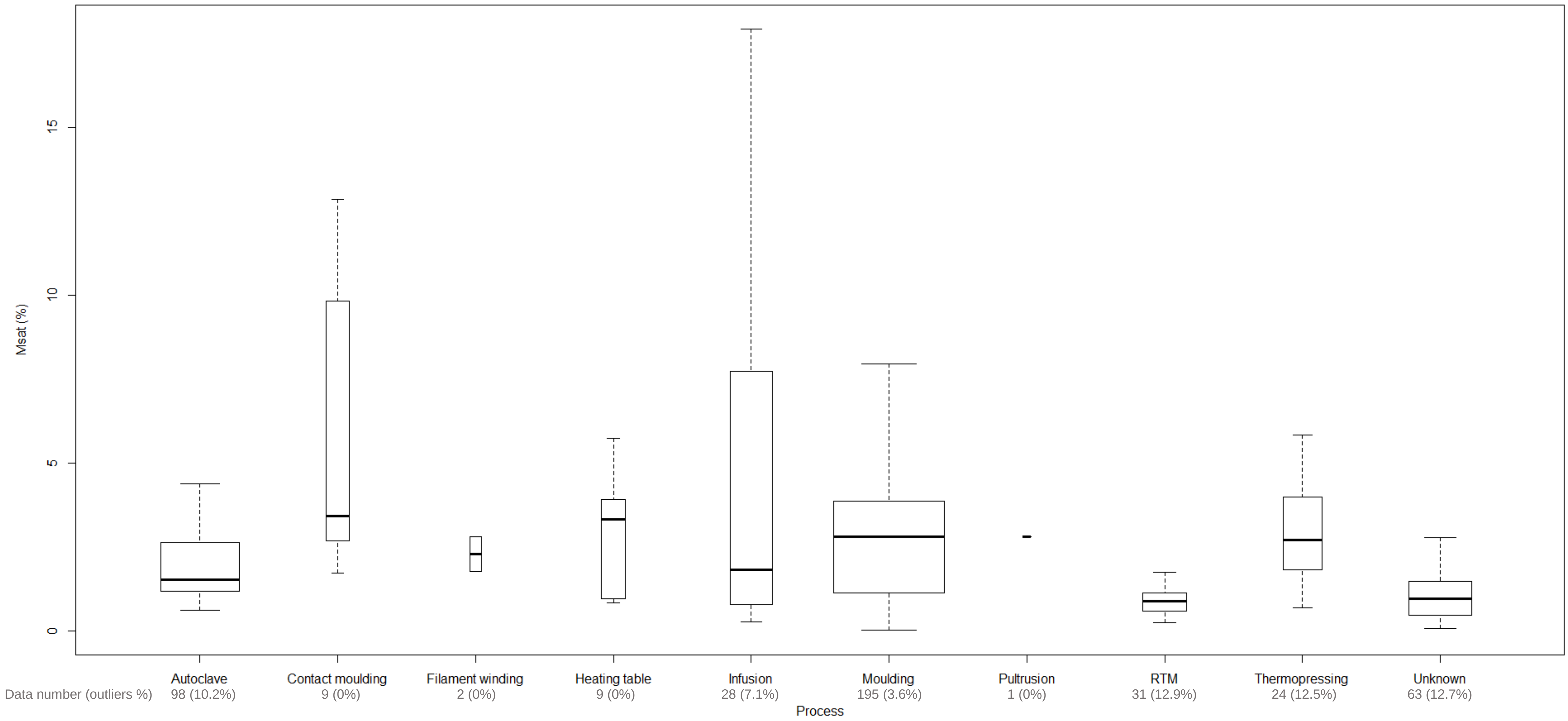

Figure S18. Boxplots of D as a function of conditioning.

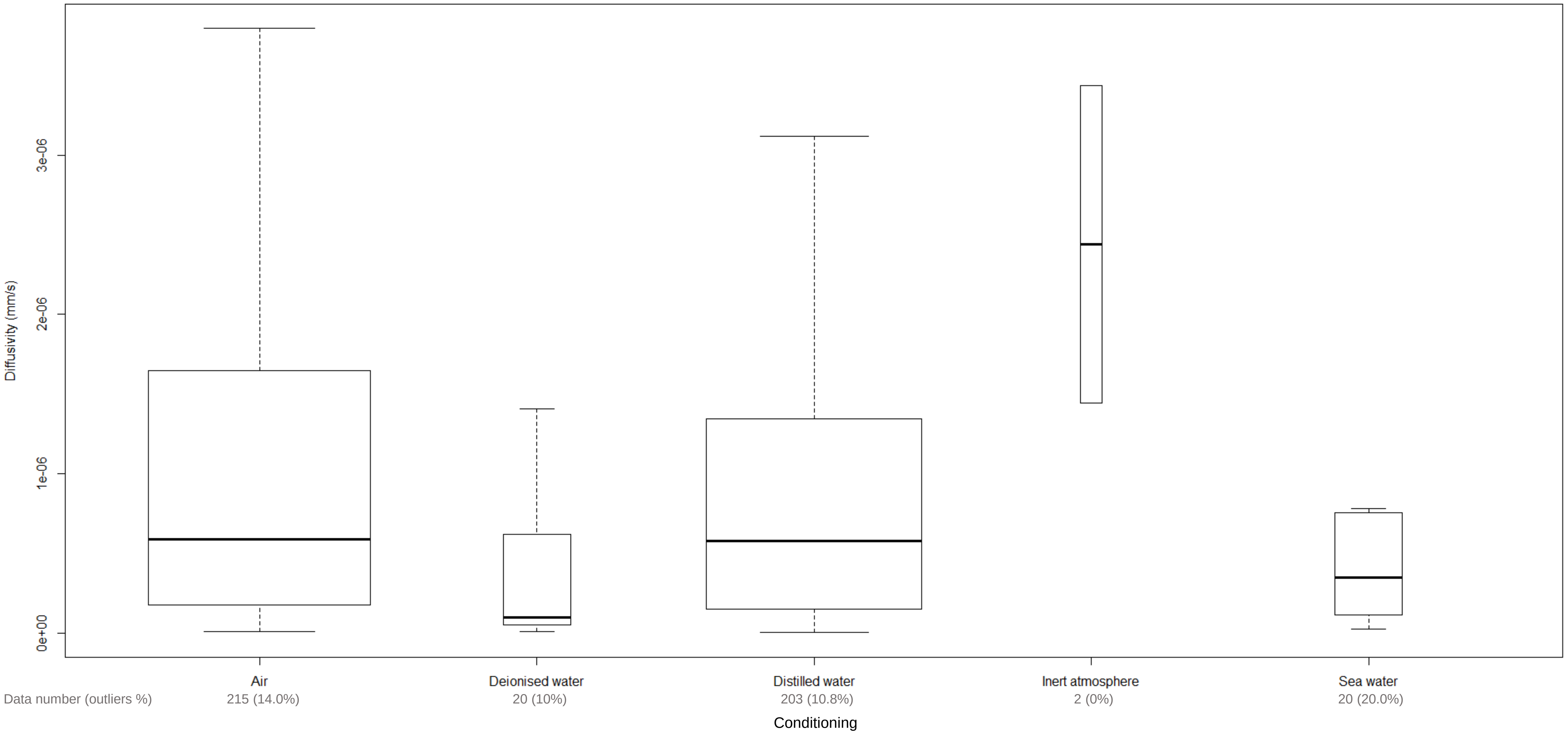

Figure S19. Boxplots of t_{sat}/h as a function of conditioning.

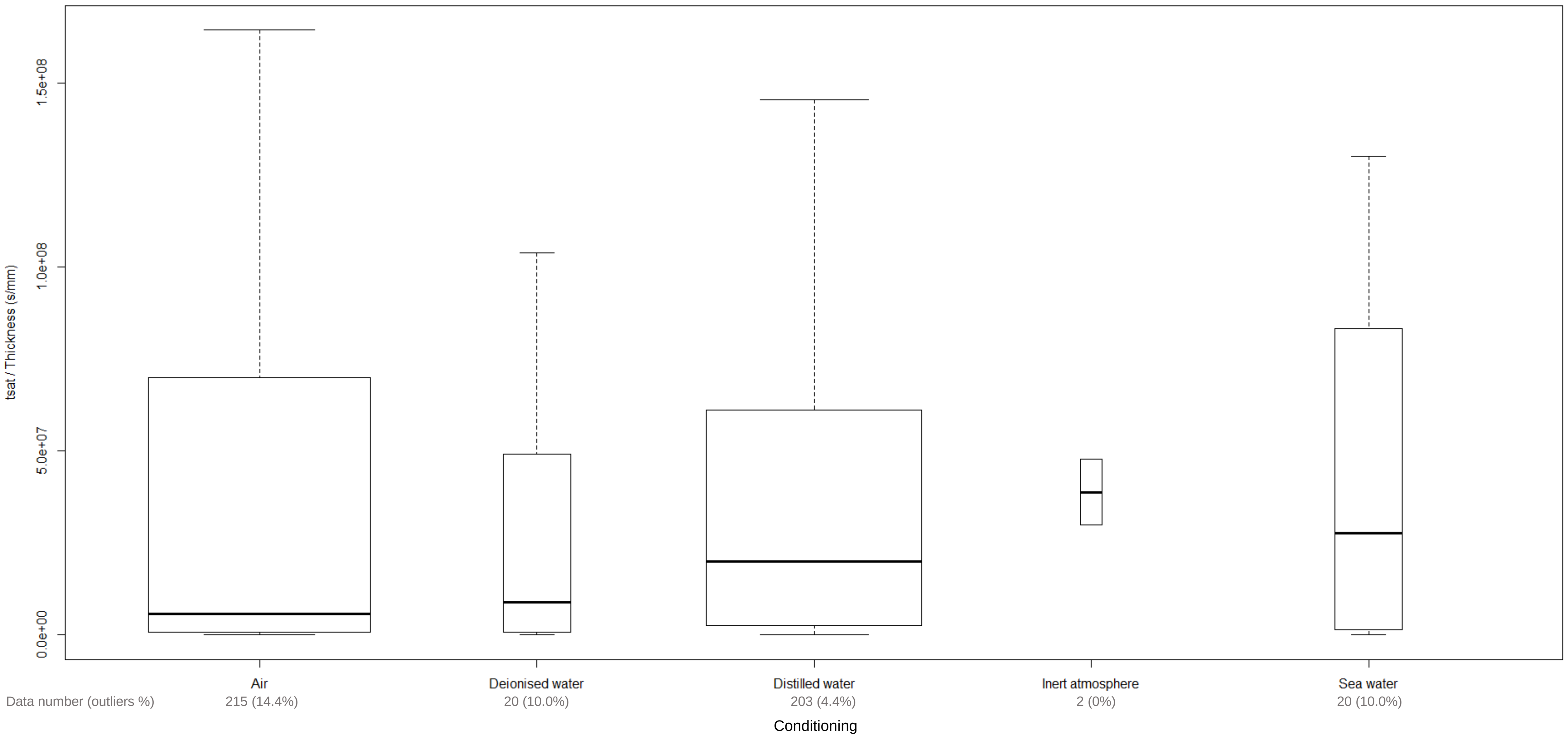

Figure S20. Boxplots of M_{sat} as a function of conditioning.

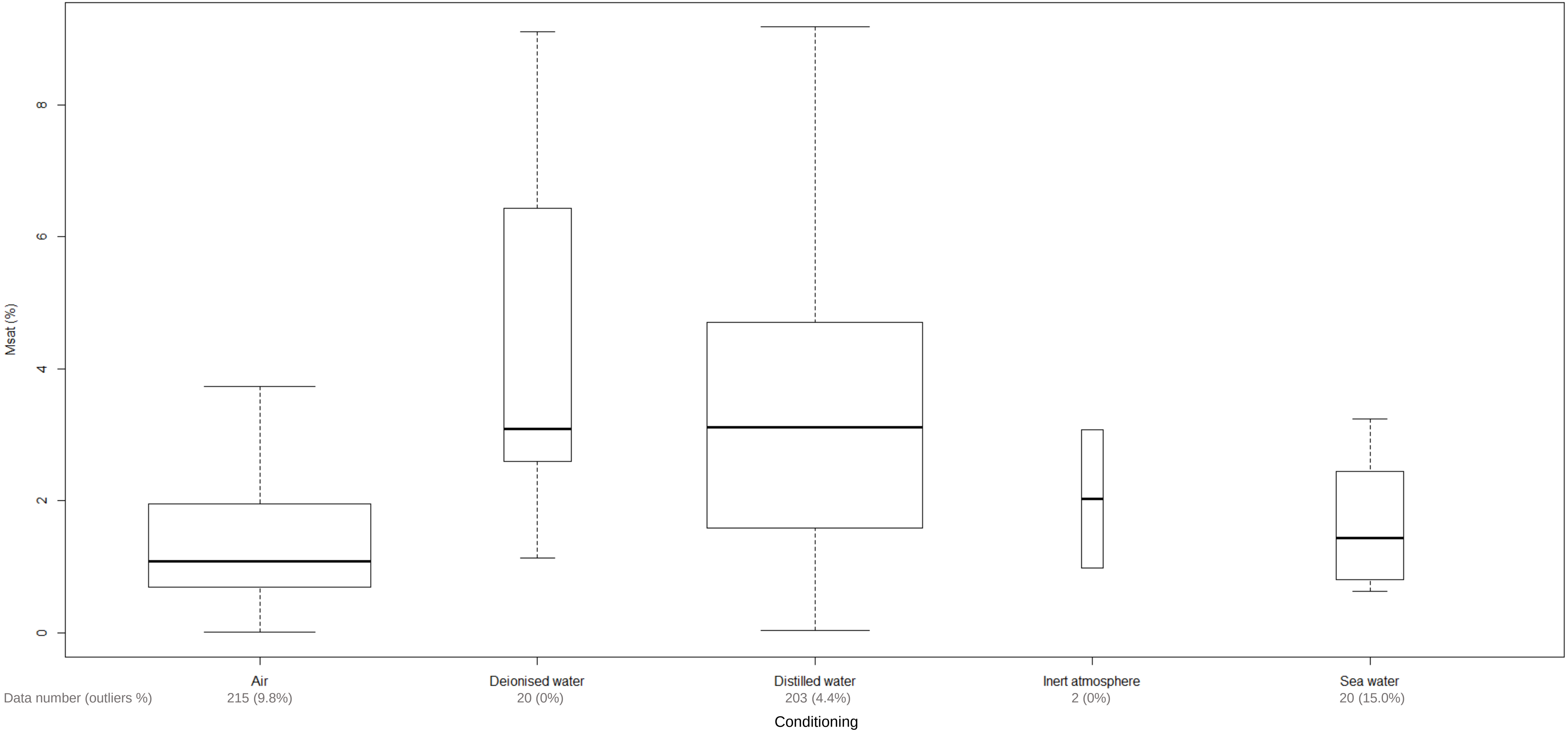

Figure S21. Fick resins' PCA: representation quality of the study variables on the principal components and correlation circles.

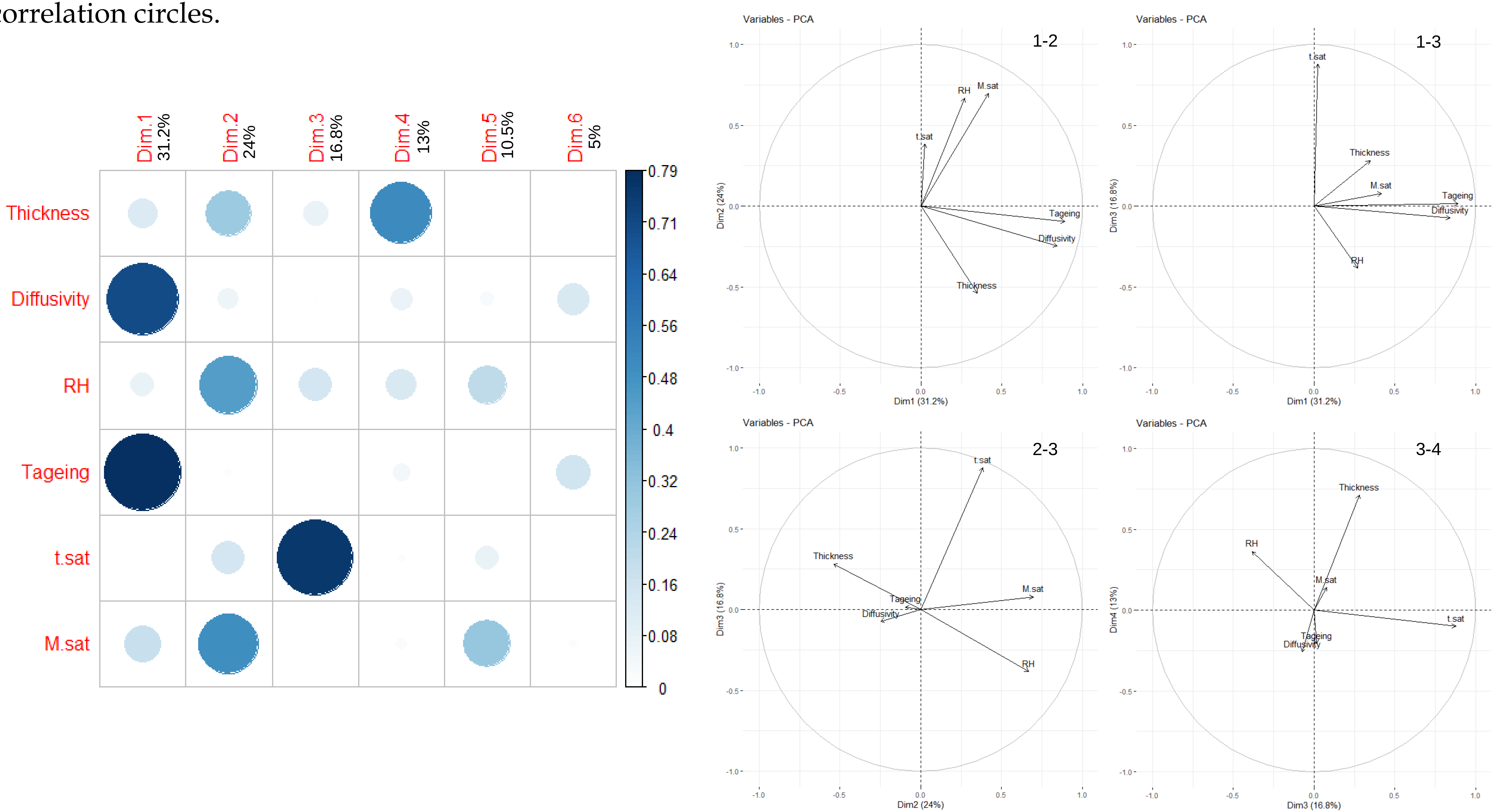

Figure S22. Fick composites' PCA: representation quality of the study variables on the principal components and correlation circles.

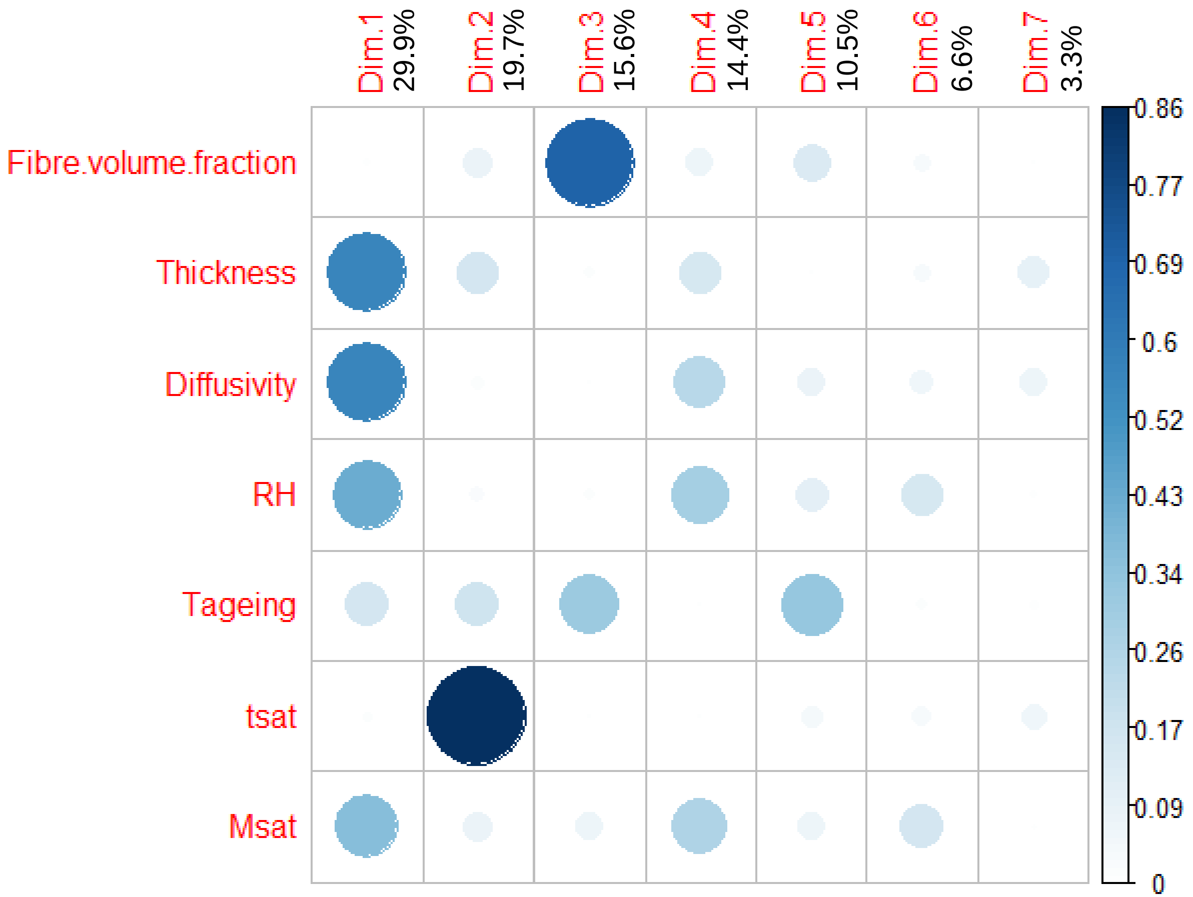

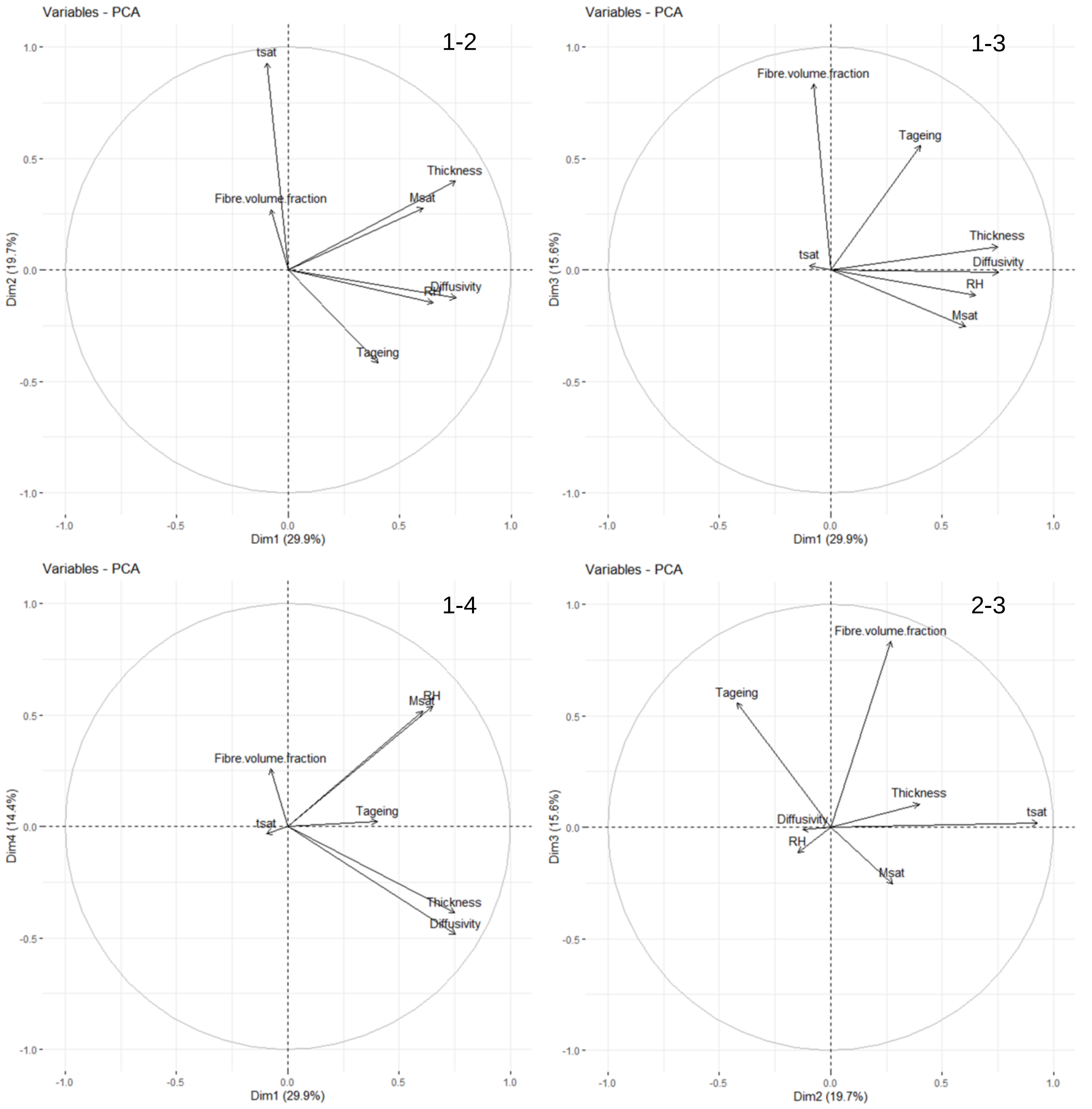

Figure S23. Dual resins' PCA: representation quality of the study variables on the principal components and correlation circles.

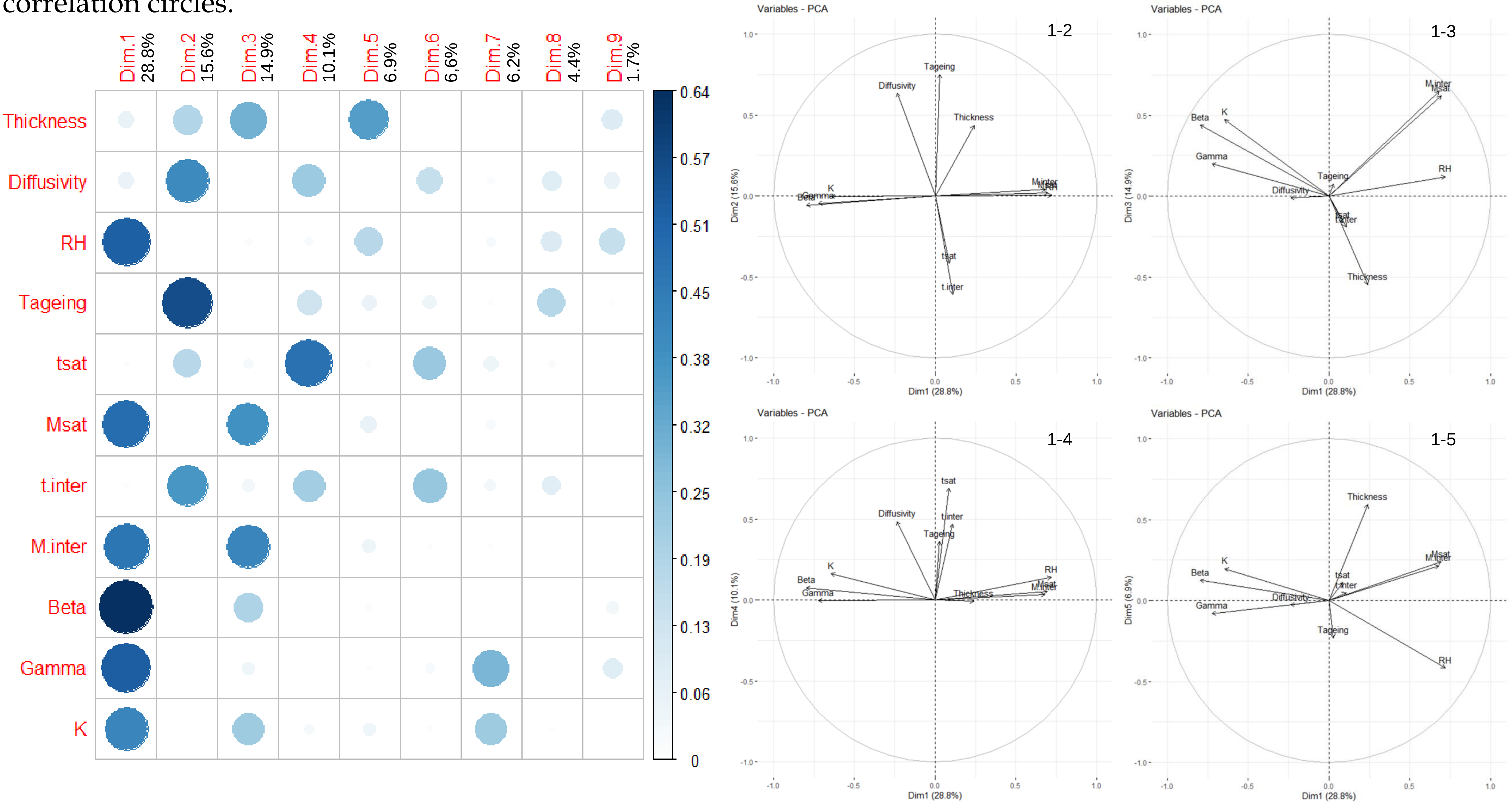

Figure S24. Dual composites' PCA: representation quality of the study variables on the principal components and correlation circles.

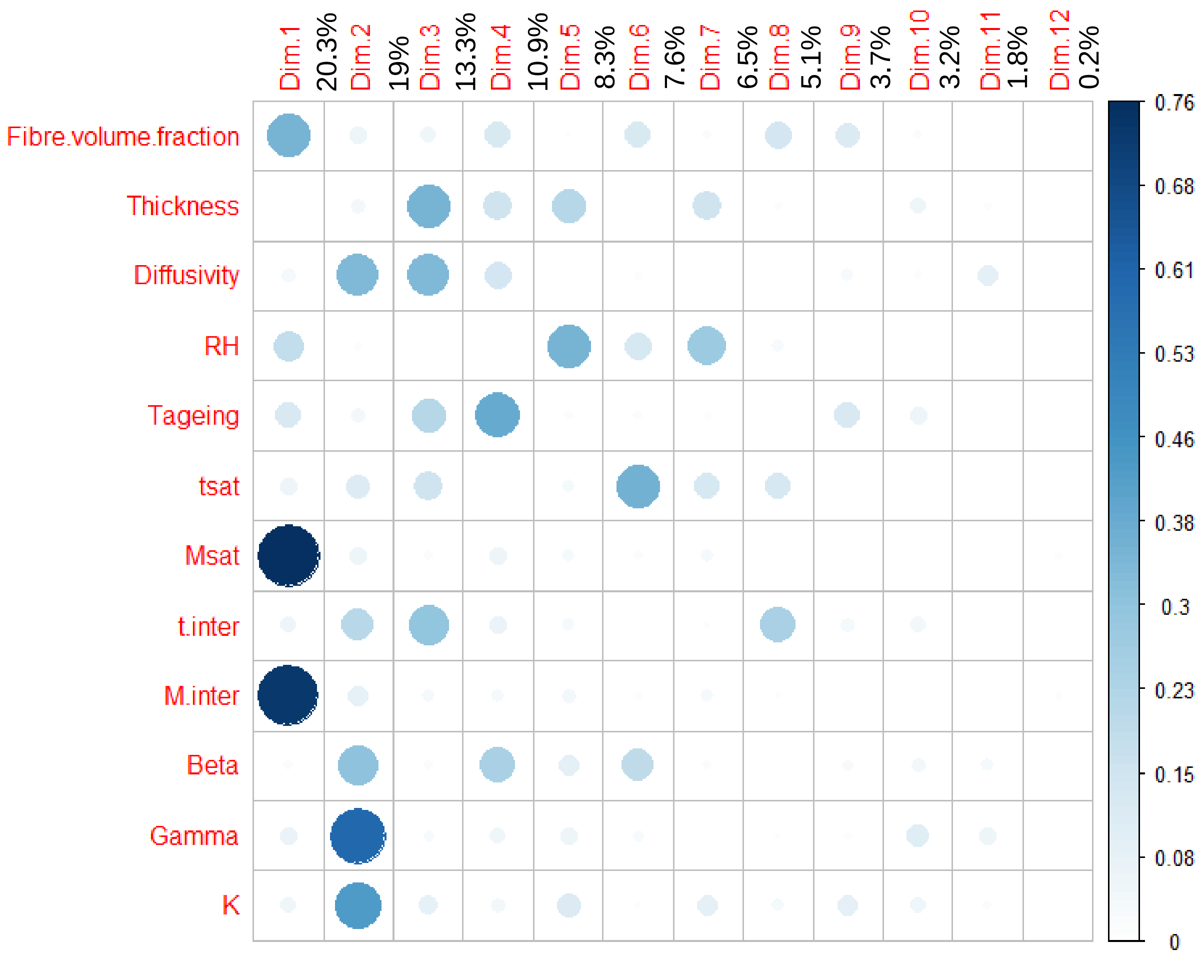

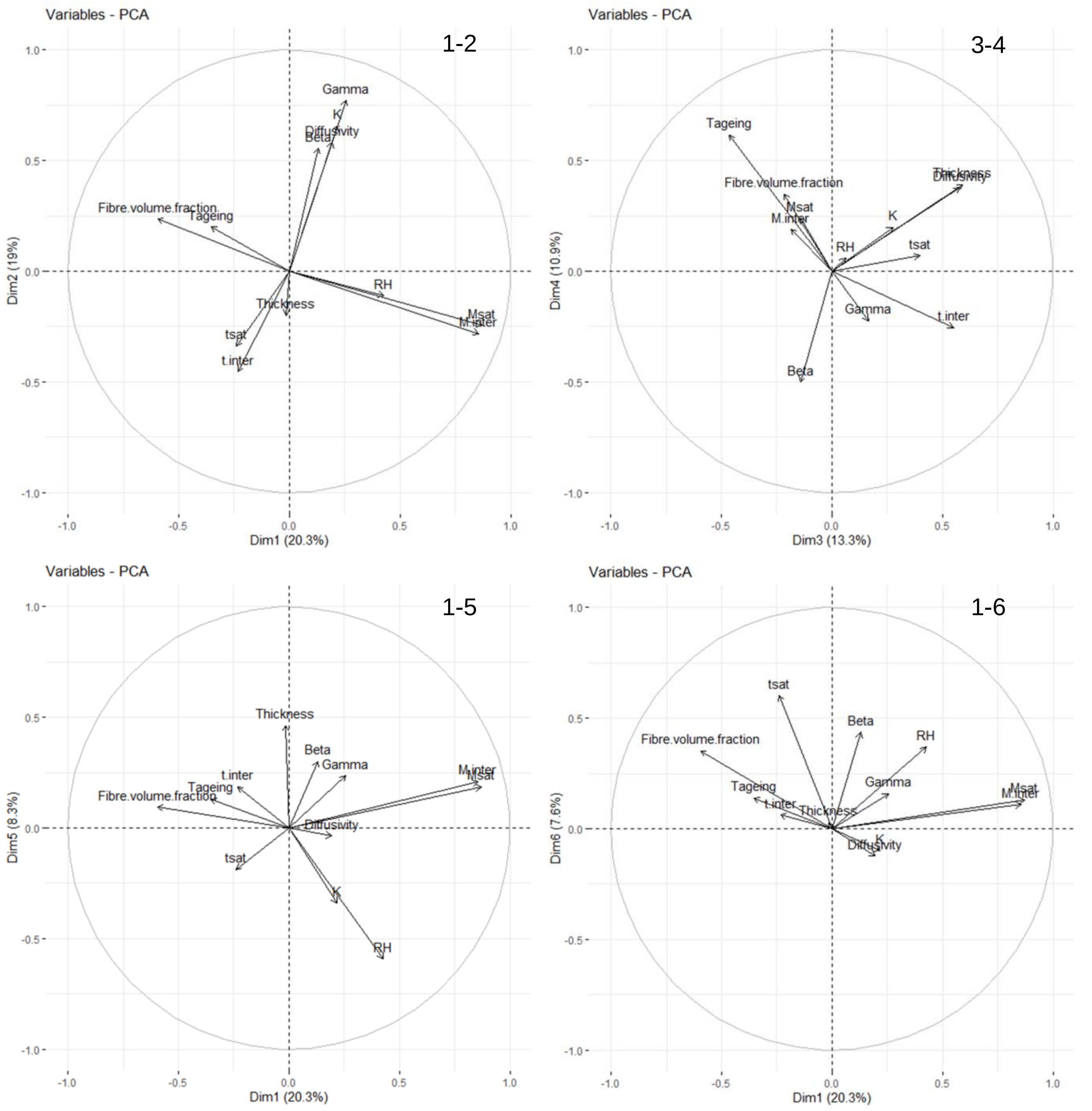

Supplement: Supplementary file 1 [file polymers-14-02832-s001.zip › polymers-1801421-supplementary.pdf]
